# Reframing the Medication Experience in Pharmacy Using Seminal Concepts of Patient-Centered Care—Implications for Practice

**DOI:** 10.3390/pharmacy9010009

**Published:** 2021-01-05

**Authors:** Brian Isetts, Anthony Olson, Jon Schommer

**Affiliations:** 1 College of Pharmacy, University of Minnesota, 308 Harvard St SE, Minneapolis, MN 55455, USA; isett001@umn.edu (B.I.); schom010@umn.edu (J.S.); 2 Essentia Institute of Rural Health, 502 E 2nd St, Duluth, MN 55805, USA

**Keywords:** patient-centeredness, medication experience, pharmacist practice

## Abstract

Team-based, Patient-Centered Care is essential to chronic disease prevention and management but there are differing ideas about the concept’s meaning across healthcare populations, settings and professions. This commentary’s objective is to empirically evaluate the theoretical relationships of the [a] Medication Experience, [b] Patient-Centeredness and other relevant component concepts from pharmaceutical care (i.e., [c] Therapeutic Relationship, [d] Patient-specific preferences for achieving goals of therapy and resolving drug therapy problems) so as to provide practice-based insights. This is achieved using a secondary analysis of 213 excerpts generated from in-depth semi-structured interviews with a national sample of pharmacists and patients about Patient-Centeredness in pharmacist practice. The four component concepts (i.e., a–d) related to the objective were examined and interpreted using a novel 3-archetype heuristic (i.e., Partner, Client and Customer) revealing common practice-based themes related to care preferences and expectations in collaborative goal setting, enduring relationships, value co-creation and evolving patient expectations during challenging medical circumstances. Most practice-based insights were generated within the Partner archetype, likely reflecting high congruence with pharmacist and patient responses related to the Medication Experience and Therapeutic Relationship. The practice-based insights may be especially useful for new practitioners and students accelerating their advancement in providing effective and efficient Patient-Centered Care.

## 1. Introduction

Cipolle, Strand, and Morley define the ‘Medication Experience’ as “the sum of all the events a patient has in (their) lifetime that involve drug therapy …which constitutes the patient’s personal experience with medications shaping his or her attitudes, beliefs and preferences about drug therapy” [1] (p. 110). This definition is directly correlated with an understanding of a “patient-centered approach” (i.e., ‘Patient-Centeredness’) in pharmacy requiring pharmacists to put the patient’s interests first and taking responsibility for all of the patient’s drug-related needs, concerns and expectations by adopting a holistic view of the patient as a person with rights, knowledge and experience that extend outside of disease, illness and pharmacotherapy [1]. The purpose of this commentary is to empirically evaluate the theoretical relationships of the Medication Experience, Patient-Centeredness and other relevant component concepts from the practice of pharmaceutical care (e.g., drug therapy problems, therapeutic relationship) so as to provide practice-based insights.

Cipolle, Strand, and Morley perceive Patient-Centeredness as a building block of the philosophy of practice in pharmaceutical care, which is the “patient-centered practice in which the practitioner assumes responsibility for the patient’s drug-related needs, is health accountable for this commitment… [and] aims to achieve positive patient outcomes…by identifying, resolving and preventing drug therapy problems.” (p. 666) [1]. They clearly articulate that the patient should always come first and that patients should drive the encounter, rather than organizing thinking, decisions and actions solely around knowledge of medications. However, the emphasis on medications and drug therapy problems reflects a contextual and often overlooked component of patient experiences and expectations (i.e., Medication Experience) that fits well with the expertise and role of the pharmacist on the healthcare team. 

The roots of Cipolle, Strand, and Morley’s understanding of Patient-Centeredness is traceable to a pair of seminal conceptualizations of the construct from the discipline of Medicine developed by Stewart and Mead and Bower, respectively [2,3,4]. Moira Stewart was a Canadian primary care physician who identified six patient-centered elements that persons desire from their physicians when communicating in primary care environments: (1) explores the patients’ experience and expectations of disease and illness (i.e., 1a. ideas about what is wrong; 1b. feelings about being ill; 1c. expectations of what should be done; 1d. the impact of the problem on daily function); (2) seeks an integrated understanding of the patients’ world (i.e., their whole person, emotional needs and life issues); (3) finds common ground on what the problem is and mutually agrees on management; (4) enhances disease prevention and health promotion; (5) the continuing relationship between the patient and the doctor; and (6) being realistic about what can be achieved [2,5,6,7].

Cipolle, Strand, and Morley’s writings on Patient-Centeredness are consistent with each of Stewart’s elements and the trio specifically highlight the first element and its four subdimensions (e.g., 1a through 1d) as necessary for a pharmacist to understand how to utilize medications in patient-centered care. They also reference the work of Shoemaker and de Oliveira for thinking about how to practice in a patient-centered manner, which specifically identifies medication-related factors impacting if and how patients take their medications such as the patient’s understandings (e.g., relevant indication, dose, frequency), feelings or attitudes (e.g., negative, positive), expectations (e.g., signs of effectiveness), concerns (e.g., side effects), behaviors (e.g., adherence) and considerations (e.g., religious, cultural, etc.) [8]. Table 1 more explicitly outlines the relationship between the Medication Experience and the first element of Stewart’s conceptualization of Patient-Centeredness with corresponding examples.

A second seminal influence of Cipolle, Strand and Morley’s conception of Patient-Centeredness that directly ties to the Medication Experience comes from Mead and Bower who identified a five component conceptualization of the construct generalizable to Medicine as a whole: (1) Biopsychosocial Perspective; (2) Patient as a Unique Person; (3) Sharing Power and Responsibility; (4) Therapeutic Alliance; and (5) Doctor as Person [3]. Each of Mead and Bower’s concepts align well with Cipolle, Strand and Morley’s understandings on Patient-Centeredness but there is an especially high degree of overlap between the former’s ‘Therapeutic Alliance’ and the latter’s ‘Therapeutic Relationship’, which is defined by Rogers as identifying shared therapeutic goals and augmenting the doctor and patient’s personal bond [9]. Both concepts refer to the bond forged from essential qualities (e.g., trust, respect, empathy, commitment, etc.) between the patient and clinician as well as a sharing of goals where the Therapeutic Relationship focuses these goals on “optimizing the patient’s medication experience” (p. 117) [1]. It is also important to note that the Therapeutic Relationship contains elements like patient responsibilities (e.g., asking questions when they arise, participating, etc.) and roles (e.g., decision-maker, teacher, the primary source of information, etc.) captured in Mead and Bower’s separate concept of ‘Shared Power and Responsibility’, but specifically identifies active engagement as a patient responsibility which is not necessary in Mead and Bower’s interpretation of the Patient-Centeredness construct [3]. This key distinction highlights that a patient-centered approach under Cipolle, Strand and Morley’s pharmaceutical care necessitates a patient-pharmacist relationship involving a concordant process of decision making reflecting a shared locus of control that generates value primarily through a co-created experience. Alternatively, Mead and Bower’s conceptualization of Patient-Centeredness provides more flexibility for the power dynamics (i.e., provider or patient locus of control) and source of value (e.g., pharmacist expertise, patient convenience) preferred and expected by patients.

## 2. Materials and Methods 

This secondary analysis drew primary data from a dissertation study examining the theoretical foundations of Patient-Centeredness in pharmacist practice using a mixed-methods, content analysis design [10] that followed Bengtsson’s content analysis approach [11]. Mixed-methods represented the best tactic for generating comprehensive and meaningful data given that Patient-Centeredness is experiential in nature and relies heavily on pharmacist and patient perspectives.

### 2.1. Primary Dataset

The primary data set was generated from semi-structured interviews with a nominated sample of patients and pharmacists spanning nine U.S. states and three types of outpatient care settings. The interviews resulted in 20 h of audio that was transcribed and converted into 439 excerpts for data analysis.

All pharmacist study participants (n = 9) were actively providing care services and had a minimum of 10,000 h of experience providing care consistent with the Joint Commission of Pharmacy Practitioners’ “Pharmacists’ Patient Care Process” (PPCP) [12]. Patient study participants (n = 6) were receiving care from pharmacists enrolled in the study and had multiple chronic conditions. The number and richness of the data sources met criteria referred to by Bengtsson and was consistent with the size and scope of most content analyses in healthcare [11]. Given the depth of the Patient-Centeredness literature and multi-factorial nature of pharmacist practices (e.g., diverse services, regulatory environments, practice models, etc.), the sample had to be sufficiently large to address the research question’s nature and transferability of the associated findings, while also small enough to enable a deep analytical dive for each respective source of data. A dense description of the primary data sources in the primary analysis from the dissertation study is presented elsewhere [10]. Trustworthiness of the qualitative data collected was assessed using methods outlined by Guba and Krefting [13,14]. Several steps were taken to preserve study participant privacy, confidentiality and anonymity and the study was reviewed and approved by the University of Minnesota, Office of the Vice President for Research, Human Research Protection Program—Institutional Review Board (IRB STUDY#00005247). In addition, the Appendix A presents definitions of terms and concepts applied in this analysis for clarity and consistency.

The procedure for data collection in the dissertation study began with the identification of potential study participants who are pharmacists using a national network of key informants known to the researcher or dissertation committee members to be a part of the development and implementation of the PPCP. This purposive sampling approach was used to efficiently identify information-rich data sources [15]. All initial contact information with potential study participants came directly from these key informants or publicly available information. A detailed description of each methodological step in the primary analysis from the dissertation study is presented elsewhere [10]. 

### 2.2. Secondary Analysis of Primary Data Subset

The richness of the primary dataset facilitated an a priori secondary analysis to provide practice-based insights for important questions related to the theoretical relationships of the Medication Experience, Patient-Centeredness and other relevant component concepts from pharmaceutical care not comprehensively addressed in the dissertation.

Among the 439 participant excerpts making up the dissertation study’s composite dataset, 213 participant excerpts (48.5%) pertained to the commentary’s objectives and were included for the secondary analysis. A mixed-methods, directed content analysis approach was used that coded excerpts with four concepts from the primary analysis: Therapeutic Relationship, Therapeutic Alliance, Medication Experience and Patient Preferences (i.e., ‘Patient-specific preferences for achieving goals of therapy and resolving drug therapy problems’). These 213 participant dataset excerpts were then analyzed to uncover common themes that could be placed in the context of specific practice-based insights and examples. 

## 3. Results

**Figure 1:**
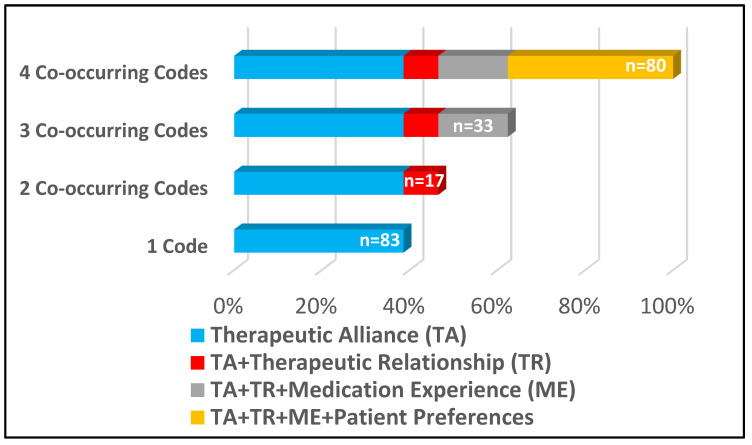
Component Concept Frequency and Co-Occurrence Distribution (N = 213).

The Therapeutic Alliance concept was coded in all excerpts included in the secondary data analysis and was followed in proportional frequency by the Therapeutic Relationship (61%), Medication Experience (53%) and Patient Preferences (38%). Figure 1 shows the proportional frequencies, revealing a non-mutually exclusive relationship most analogous to stacking dolls where less frequently coded concepts are nested or combined with the next most frequently coded concept. For instance, the least frequently coded concept of Patient Preferences is only found when co-occurring (i.e., never found alone) with the three other concept codes (bar titled “4 Co-occurring codes”). Similarly, the second least frequently coded concept of the Medication Experience is only present in conjunction with the Therapeutic Relationship and Therapeutic Alliance, and the Therapeutic Relationship is only present when co-occurring with the Therapeutic Alliance. Only the Therapeutic Alliance code was found independent of the other concepts in the analysis. 

**Figure 2:**
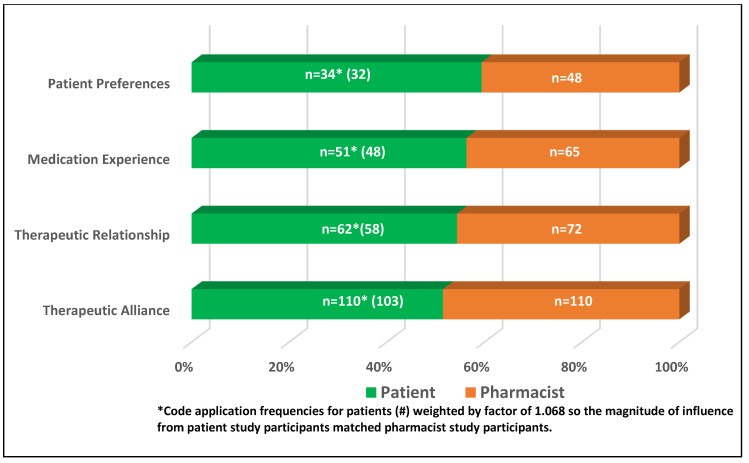
Adjusted concept distributions by participant type (N = 213).

Of the 213 excerpts included in this secondary analysis, 110 of the data excerpts came from pharmacist study participants (51.5%), with the remaining 103 from patient study participants (48.5%). Figure 2 displays the adjusted proportional frequency for each component concept by participant type, with patient concept codes weighted by a factor of 1.068 to equalize the magnitude of influence from patient study participants and pharmacist study participants.

## 4. Discussion

There are a number of noteworthy practice-based contributions and insights related to patient preferences and expectations in pharmacist care that are generated from this secondary analysis. The dissertation study titled ‘*Patient-Centeredness in Pharmacist Practice: Filling a foundation for what counts to patients*’ [10] provides a useful 3-archetype framework for discussing these findings: ‘Partner’, ‘Client’, and ‘Customer’. The Partner archetype represents a patient and pharmacist relationship where care preferences and expectations are characterized by a concordant process of decision making that generates value primarily through a co-created experience. The Client archetype refers to a relationship between the patient and pharmacist where preferences and expectations align most with fiduciary contours and where the predominant value comes primarily from specialized expertise individualized to key characteristics of the patient by the pharmacist. Finally, the Customer archetype consists of preferences and expectations surrounding the patient and pharmacist relationship that are transactional in nature and primarily valued in terms of convenience for the patient. Additional detail about the archetypes and their development can be found elsewhere (pp. 107–108, 156–176) [10].

As can be expected, most practice-based insights were generated within the Partner archetype given the methods employed for this secondary content analysis investigation focusing on pharmacist and patient responses related to the Therapeutic Relationship, Therapeutic Alliance, Medication Experience and Patient Preferences.

### 4.1. Collaborative Goal Setting within the Therapeutic Relationship

A classic example of how important shared decision-making and collaborative goal setting is within the Therapeutic Relationship is described in observations from a study participant with newly diagnosed hypertension who was adamant about an initial goal of doing whatever it takes to avoid the use of medications. In this case, the pharmacist conveyed all the morbidity and mortality benefits of controlling blood pressure, while helping to establish interventions and plans to improve physical activity, diet and other lifestyle changes. Although this patient did initiate pharmacotherapy a few month later, the pharmacist and patient data excerpts illustrated an open and honest, back and forth relationship implying whereas much time was invested in exploring and better realizing the contributions of the other party, as was arriving at options or recommendations. This example highlights that a sufficient amount of time and a conducive space is required for this kind of communication. This example also sheds light on an unsubstantiated misconception of Patient-Centered Care that it takes too much time as research suggests that care with patients preferring a dialogue with their provider is more time and cost-efficient because it better identifies and supports the fulfillment of priorities and goals that are complex in nature [16].

### 4.2. Longevity of Care Factor in Enduring Relationships

Another thread of the Partner archetype relates to a longevity of care factor illustrating an enduring relationship between the patient and pharmacist built on years of interaction through periods of successful and challenging management of a patient’s health conditions and drug therapies. One study participant noted that they choose to drive over an hour to get care from a pharmacist they’ve worked with for many years despite there being other pharmacists providing similar services that are closer in distance to them. One reason cited among study participant statements within the Partner archetype explaining this enduring longevity is that patients feel the care they receive from their pharmacist is unique and possibly even irreplaceable.

A unique care customization factor was also evident in the Partner archetype exemplified by study participant statements displaying care preferences for not only a specific pharmacy but also a specific pharmacist. Patients that display care preferences at the level of partnership have found and value a level of tailoring they do not think they can get anywhere else.

### 4.3. Co-Created Value Experience

One other important characteristic of the Partner archetype relates to a co-created experience factor. The patient receives value from having a pharmacist who is invested, sharing and a co-creator of their medication experience. Value co-creation refers to the creation of value through the interaction between the provider and recipient of a service [17]. This co-production of value is a co-equal collaborative process between the patient and pharmacist to develop a care plan that optimally addresses the priorities, goals and preferences for care. This preference and expectation for a co-created experience” in the Partner archetype is embodied in both patient and pharmacist responses related to mutual respect and trust for the expertise of the other, the patient’s expertise in their own life (e.g., motivators, resources, challenges, etc.) and the pharmacist’s expertise in navigating the healthcare system and optimizing medications for safety, effectiveness and accessibility. Patients may or may not have relationships resembling the Partner archetype with other providers in the healthcare team depending on their preferences, expectations and value involving care. Similarly, some patients may have relationships resembling the Partner archetype with other members of the healthcare team but not with the pharmacist. This speaks to the importance of coordination and role diversity in team-based approaches to providing Patient-Centered Care.

### 4.4. Evolving Expectations during Challenging Healthcare Circumstances

Although this secondary investigation focusing on pharmacist and patient responses related to the therapeutic relationship, therapeutic alliance, medication experience and patient-specific preferences for achieving goals of therapy and resolving drug therapy problems produced few results in the Client and Customer archetypes, there were important practice-based insights revealed in terms of patients’ transitions of care, new diagnoses and changing clinical status. Practitioners need to be cognizant of sudden changes in patient expectations and preferences in the face of new and challenging medical circumstances. The patient-pharmacist relationship can quickly evolve from one characterized by the Partner to the Client archetype. For instance, when an individual is informed that they have a metastatic carcinoma diagnosis they can become overwhelmed with the amount and level of information needed to process treatment decisions. In this instance, patients and caregivers may be less inclined to engage in co-creation and shared decision-making, deferring to a relationship more consistent with the Client archetype. This may be seen more frequently when patients are facing circumstances where they feel vulnerable or ill equipped to manage without the technical expertise, skill or resources of the fiduciary. It is very important to point out that when individuals do experience sudden transitions of care that may evolve into a Client archetype, a practitioner’s philosophy of practice related to establishing a Therapeutic Relationship characterized by trust, empathy, respect, authenticity and responsiveness takes on an equal, if not greater imperative [1].

### 4.5. Limitations

For experienced practitioners, some of the practice-based insights discussed in this secondary analysis may seem like second nature. It’s what we do every day as seasoned practitioners that appears to happen almost effortlessly. However, for new practitioners and students these insights may serve as a pathway to concepts and approaches that help them more efficiently and effectively advance in becoming experienced practitioners providing high quality care across diverse circumstances.

Another limitation of this secondary investigation resides in the transferability of these practice-based insights given the nature of content analysis limits the size and ability to randomize samples. However, one of the benefits of this secondary analysis is in translating the results of a very comprehensive and highly complex dissertation study into meaningful practice insights less achievable in purely quantitative studies that new practitioners and the profession can utilize to accelerate progress toward more effective and efficient Patient-Centered Care.

## 5. Conclusions

Patient-Centeredness is believed to be integral to team-based care leading to successful prevention and management of chronic disease as well as the promotion of health and well-being. This secondary investigation employed content analysis methods focusing on pharmacist and patient responses related to the Therapeutic Relationship, Therapeutic Alliance, Medication Experience and patient-specific preferences for achieving goals of therapy and resolving drug therapy problems (i.e., Patient Preferences) for the purpose of providing practice-based insights that new practitioners and students can use to accelerate progress toward becoming more effective, efficient and compassionate practitioners. Although the Partner archetype of patient-pharmacist relationships for Patient-Centeredness preferences and expectations of care dominated the practice-based implications, this secondary analysis revealed that the Client and Customer archetypes of Patient-Centeredness in pharmacist care provide insights into the amount and level of information that patients can assimilate in shared decision-making to achieve goals of therapy and resolve drug therapy problems during challenging health care and medication use experiences.

## Figures and Tables

**Table 1:** Links and examples of Stewart’s Patient-Centered Care Sub-Dimensions and aspects of the Medication Experience (adapted from Cipolle, Strand, and Morley) [1].

CSP’s Medication-Related Applications of Stewart’s Four Patient-Centered Subdimensions	Corresponding Aspect of the Medication Experience	Examples
The patient’s ideas/meaning about their medication	Understandings, Concerns, Adherence	Symbol of a permanent loss of health A chance to gain understanding A conduit to find support or dependency Part of a normal progression in life A consequence of past choices
The patient’s feelings about taking medication	Understandings, Expectations/Attitudes, Adherence	Anxiety over what medication means Reprieve from roles or responsibilities due to taking the medication Anger about why they need to take medication Guilt about how managing their medications impact others in patient’s life
The patient’s expectations of what should be done by the clinician	Expectations	Prescription drug An understanding of the medication Clearly answer questions Listen Give advice Work together to create a plan
The patient’s perceived impact of taking medication on their daily function	Understandings, Concerns, Adherence	An impediment or change to patient’s day to day life Modification of relationship dynamics in the patient’s life

Note: CSP = Cipolle, Strand and Morley.

## Data Availability

The data presented in this study are available on request from the corresponding author. The data are not publicly available due to University of Minnesota–Institutional Review Board policies.

## Appendix A. Definitions and Terms

For clarity and consistency, the following terms applied in this commentary are defined as follows:

- Patient-Centeredness—refers to intangible concepts like philosophy, values or principles (e.g., dignity, respect, personalization, etc.) that underlie and inform Patient-Centered Care [18,19].
- Patient-Centered Care—refers to the concrete activities, behaviors, practices, technical interventions and health systems innovations in service of Patient-Centeredness (e.g., self-management support, shared decision making, motivational interviewing, etc.) [18,19].
- Construct—a highly abstract term that is defined by less abstract terms (i.e., concepts) and inferred from observable phenomena [20].
- Concept—a vivid picture of something which helps to understand the category and diversity of a construct [20].
